# New tridecapeptides of the theonellapeptolide family from the Indonesian sponge *Theonella swinhoei*

**DOI:** 10.3762/bjoc.9.188

**Published:** 2013-08-13

**Authors:** Annamaria Sinisi, Barbara Calcinai, Carlo Cerrano, Henny A Dien, Angela Zampella, Claudio D’Amore, Barbara Renga, Stefano Fiorucci, Orazio Taglialatela-Scafati

**Affiliations:** 1 Dipartimento di Farmacia, Università di Napoli “Federico II”, via D. Montesano 49, 80131 Napoli, Italy; 2 Dipartimento di Scienze della Vita e dell’Ambiente, Università Politecnica delle Marche, Via Brecce Bianche, 60131 Ancona, Italy; 3 Faculty of Fishery and Marine Science, Sam Ratulangi University, Manado, Indonesia; 4 Dipartimento di Medicina Clinica e Sperimentale, Università di Perugia, Via Gambuli 1, 06132 Perugia, Italy

**Keywords:** antiproliferative activity, cyclic peptides, marine metabolites, theonellapeptolides, 2D NMR

## Abstract

Chemical analysis of the organic extract of *Theonella swinhoei* yielded two new tridecadepsipeptides of the theonellapeptolide family, namely sulfinyltheonellapeptolide, characterized by a methylsulfinylacetyl group at the *N*-terminus, and theonellapeptolide If, the first member of this class of compounds to show four valine residues. The structures of the compounds, isolated along with the known theonellapeptolide Id, were determined by extensive 2D NMR and MS/MS analyses followed by application of Marfey’s method. The isolated peptides exhibited moderate antiproliferative activity against HepG2 cells, a hepatic carcinoma cell line.

## Introduction

Three decades of extensive chemical investigation [1] have clearly evidenced that marine sponges of the genus *Theonella* (Lithistida, Theonellidae) are treasure troves of secondary metabolites. The chemical diversity of the isolated compounds ranges from unusual steroids (exemplified by the 4-methylene sterol theonellasterol [2–3] and truncated side-chain sulfated steroids [4]), to complex macrocyclic polyketides (as the well-known swinholide A, now a reference compound in the class of actin interacting cell growth inhibitors [5]), polyene derivatives (as aurantosides [6]), and polypeptides/depsipeptides. The biosynthesis of several secondary metabolites of *Theonella* has been ascribed to symbiotic microorganisms, as in the case of the polyketide onnamide [7] and the polypeptide polytheonamide [8]. It is, however, not unreasonable to presume that a symbiotic role in the production of secondary metabolites could be crucial in many other cases.

Probably the most distinctive class of secondary metabolites of *Theonella* is given by complex polypeptides whose uncommon amino acids have been postulated to have either nonribosomal (NR) or post-translationally modified ribosomal (PMR) origin [8]. Several classes of *Theonella* polypeptides have been isolated to date, and they often show peculiar features such as largely rearranged amino acidic units, either D- or L-configurations at the α-carbons, and the formation of macrocycles through amide or ester bonds. To categorize the plethora of *Theonella* polypeptides we could identify at least eight structural types: theonellamides (glycosylated imidazole-containing macrocycles) [9], keramamides (including oxazole or thiazole rings) [10], papuamides (HIV inhibitory macrocyclic depsipeptides) [11], polytheonamides (cytotoxic linear polypeptides) [12], cyclotheonamides (thrombin and serine protease inhibitors) [13], perthamides (anti-inflammatory cyclopeptides) [14–15], solomonamides [16] and theonellapeptolides (cyclic tridecapeptides including several *N*-methylated and D-amino acids) [17].

**Figure 1:**
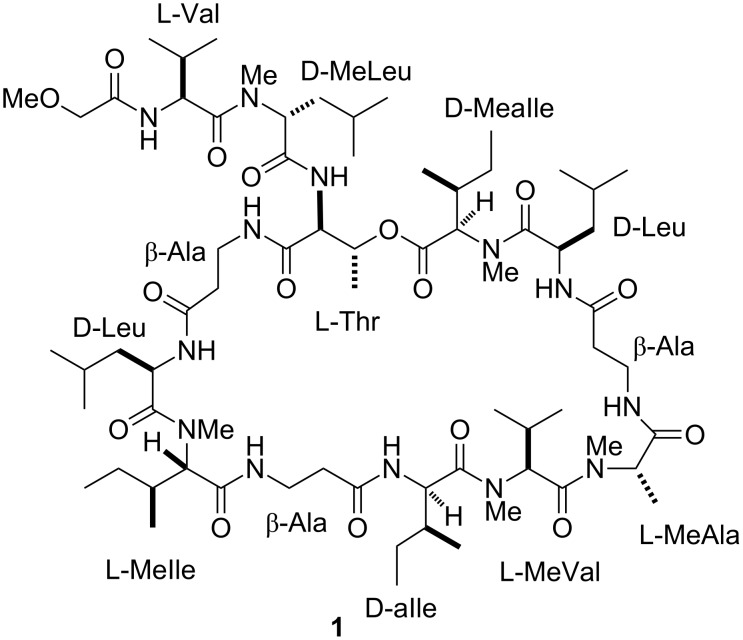
Structure of the known compound theonellapeptolide Id (**1**).

**Figure 2:**
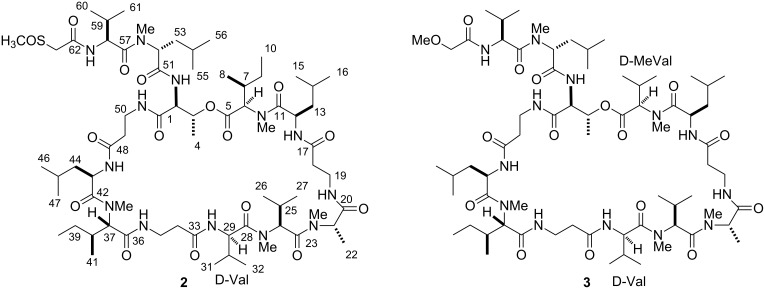
Structures of sulfinyltheonellapeptolide (**2**) and theonellapeptolide If (**3**).

In the context of our long-standing interest for bioactive secondary metabolites from marine invertebrates, we have recently investigated a specimen of *T. swinhoei* collected off the coasts of Manado (North Sulawesi, Indonesia), which proved to be rich in aurantosides [6] and 4-methylene steroids [18], while polyketide macrolides and peptide-based derivatives were extremely rare if not absent. Remarkably, the chemical analysis of a different specimen of *T. swinhoei*, collected in the same area as the previous one, exhibited an extremely different secondary metabolite composition. In particular, this second specimen has been found to be rich in polypeptides and, from the CHCl_3_ phase of the organic extract, we have identified the major member of this class as theonellapeptolide Id (**1**) (Figure 1). In this paper we report the results of the chemical analysis of the polypeptide-containing fraction, which resulted in the isolation of two new theonellapeptolides, namely sulfinyltheonellapeptolide (**2**) and theonellapeptolide If (**3**) (Figure 2). We describe the structural elucidation of these new metabolites and the results of the preliminary pharmacological evaluation of the isolated compounds on HepG2 cells (hepatocarcinoma cell line).

## Results and Discussion

*Theonella swinhoei* was collected in the Bunaken Marine Park of Manado (North Sualwesi, Indonesia) in January 2010 and frozen immediately after collection. The frozen material was repeatedly extracted with methanol, and the crude extract was subjected to a modified Kupchan’s partitioning procedure [19] to obtain *n*-hexane, CHCl_3_ and *n*-BuOH extracts. The CHCl_3_ extract was chromatographed by silica gel MPLC, and fractions eluted with CH_2_Cl_2_/MeOH 97:3 were further purified by HPLC on Nucleodur or Ascentis reversed-phase columns (eluent MeOH/H_2_O 87:13) to obtain the known theonellapeptolide Id (**1**, 68.5 mg), and the new analogues sulfinyltheonellapeptolide (**2**, 4.6 mg) and theonellapeptolide If (**3**, 2.1 mg) (Figure 2). The known compound was identified on the basis of the comparison of its spectroscopic data with those published in the literature [17].

Theonellapeptolides are tridecapeptide lactones characterized by the presence of aliphatic and non-polar amino acids including high ratio of D-amino acids, *N*-methyl amino acids, and β-amino acids. In particular, the structure of theonellapeptolide Id (**1**) includes three β-Ala, three D-Leu (one *N*-methylated), two L-Val (one *N*-methylated), one L-MeAla, one L-Thr, one L-MeIle, and two D-alloIle (one *N*-methylated) units.

**Figure 3:**
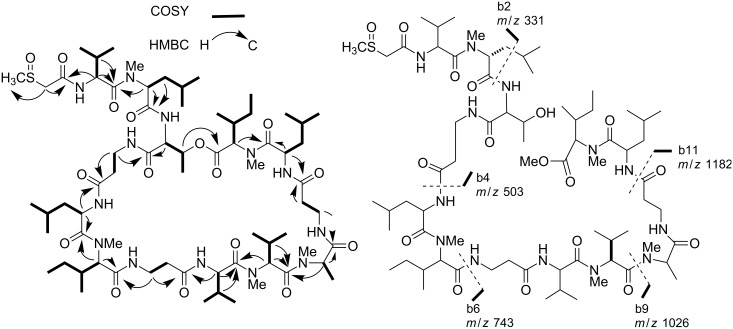
COSY and key HMBC correlations (left) and MS/MS fragmentations of **2** and its ring-opened methanolysis product, respectively.

Sulfinyltheonellapeptolide (**2**, [α]_D_ −38.1, *c* 0.1, MeOH) was isolated as a colorless amorphous solid with pseudomolecular ion peaks at *m*/*z* 1423 [M + H]^+^ and 1445 [M + Na]^+^ in the ESIMS, and high-resolution analysis established the molecular formula C_69_H_123_N_13_O_16_S. Inspection of its ^1^H NMR spectrum (CD_3_OD, Table 1) clearly suggested the peptide nature of **2** and placed it into the theonellapeptolide class. Given this characterization of **2** the presence of the sulfur atom in the molecular formula appeared especially remarkable. In particular, the ^1^H and ^13^C NMR data of **2** indicated the presence of six methyl singlets (δ_H_ 2.77, 2.79, 3.22, 3.30, 3.31, and 3.35) and fourteen carbonyl groups (resonating from δ_C_ 166.5 to 177.0). Extensive analysis of homonuclear and heteronuclear 2D NMR spectra including ^1^H/^1^H COSY, ^1^H/^1^H TOCSY, ^1^H/^13^C HSQC and ^1^H/^13^C HMBC allowed us to overcome the difficulty posed by the severe signal overlap in the ^1^H NMR spectrum and to establish the presence of three β-Ala, three Leu (one *N*-methylated), two Ile (both *N*-methylated), three Val (one *N*-methylated), one Thr, and one *N*-methylAla, the same amino acid composition as in theonellapeptolide Id (**1**) except for one residue. The above analysis left unassigned a methyl singlet at δ_H_ 2.79 (δ_C_ 39.2) and a pair of mutually coupled doublets at δ_H_ 3.67 and 3.86, with these latter signals showing HMBC correlations both with the methyl carbon at δ_C_ 39.2 and an amide carbonyl resonating at δ_C_ 166.5. The ^1^H and ^13^C NMR chemical shift values of both the methyl and the methylene groups strongly suggested their linkage to a sulfoxide group, and the presence of a methylsulfinylacetyl (MeS(O)Ac) subunit was also in full agreement with the molecular formula. The amino acid sequence of **2** was then disclosed by careful analysis of the pattern of HMBC correlations, whose key cross-peaks are shown in Figure 3. The cross-peak of the Thr low-field shifted β-CH (δ_H_ 5.19) with the Me-Ile carbonyl group revealed the presence of an ester linkage.

**Table 1:** ^1^H (500 MHz) and ^13^C (125 MHz) NMR data of sulfinyltheonellapeptolide in CD_3_OD (**2**).

AA	Position	δ_H_, m, *J* in Hz	δ_C_	AA	Position	δ_H_, m, *J* in Hz	δ_C_

L-Thr	1	–	171.2	β-Ala	33	–	173.2
	2	4.36, m	58.5		34/34’	2.29, m; 2.59, m	36.5
	3	5.19, m	70.6		35/35’	3.10, m; 4.23, m	36.8
	4	1.11, d, 6.5	19.1	L-Me-Ile	36	–	172.5
D-Me-Ile	5	–	173.4		37	3.14, m	71.2
	6	5.06, m	62.5		38	2.48, m	36.5
	7	2.14^a^	34.2		39/39’	1.04, m; 1.92^a^	30.2
	8	0.78, d, 7.2	11.5		40	1.00^a^	13.2
	9/9’	1.10, m; 1.38, m	26.5		41	0.81, d, 6.4	15.3
	10	0.98, t, 7.6	17.0		37-NMe	3.35, s	39.9
	6-NMe	3.22, s	32.0	D-Leu	42	–	176.2
D-Leu	11	–	177.0		43	5.06, m	50.6
	12	5.03, m	50.5		44	1.72^a^; 1.25^a^	41.2
	13/13’	1.74^a^, 1.25^a^	40.4		45	1.80^a^	26.6
	14	1.81^a^	26.3		46	1.03^a^	21.0
	15	0.89^a^	11.1		47	1.04^a^	25.0
	16	0.94 d, 7.3	17.2	β-Ala	48	–	175.0
β-Ala	17	–	174.3		49/49’	2.28, m; 2.36, m	39.1
	18/18’	2.23, m 2.29 m	39.5		50/50’	3.14, m; 3.81, m	37.7
	19/19’	3.24, m 3.69, m	38.2	D-Me-Leu	51	–	175.2
L-Me-Ala	20	–	172.5		52	5.24, m	57.5
	21	5.17^a^	58.5		53/53’	1.41^a^; 2.03 m	39.5
	22	1.45, d, 7.1	16.0		54	1.52^a^	27.1
	21-NMe	2.77 s	30.5		55	0.83, d, 6.7	22.1
L-Me-Val	23	–	173.0		56	0.95^a^	24.3
	24	5.02^a^	59.4		52-NMe	3.30 s	33.0
	25	2.40, m	30.2	L-Val	57	–	176.0
	26	0.86^a^	20.6		58	4.76, m	57.7
	27	0.92, d, 6.1	20.8		59	2.12, m	32.5
	24-NMe	3.31 s	31.9		60	0.96^a^	20.4
D-Val	28	–	173.2		61	1.01^a^	19.2
	29	4.76, m	68.9	Me-SAc	62		166.5
	30	2.68, m	27.6		63/63’	3.67, d, 11.2;	58.9
	31	0.86^a^	18.1			3.86, d, 11.2	
	32	1.17, d, 6.5	23.3		63-SOMe	2.79, s	39.2

^a^Overlapped with other signals.

HMBC analysis also revealed that the amide carbonyl at δ_C_ 166.5 assigned to the methylsulfinylacetyl subunit was correlated to the Hα of Val-1 residue, thus establishing the acetylation of the *N*-terminus in **2**. Methanolysis of **2**, followed by ESI (positive ion mode) MS/MS analysis of the obtained acyclic methyl ester derivative provided key fragment peaks, shown in Figure 3, giving definitive confirmation of the sulfinyltheonellapeptolide gross structure. In addition to the pseudomolecular ion at *m*/*z* 1454 [M + H]^+^, corresponding to the introduction of 32 mass units (MeOH) in the molecule, the ESIMS/MS spectrum provided several fragment ion peaks corresponding to *N*-terminus fragments due to the cleavage of the amide bond, and referred to as the b series in Roepstorff and Fohlman nomenclature [20]. In particular, the presence of the b2 fragmentation peak at *m*/*z* 331 supported, once again, the presence of the MeS(O)Ac unit.

Complete acid hydrolysis of **2** and Marfey’s analysis [21] on the hydrolysate (derivatization with L-FDAA, 1-fluoro-2,4-dinitrophenyl-5-L-alanine amide, followed by LC–MS comparison with the FDAA derivatives of appropriate standards) enabled us to determine the configuration of the chiral amino acid residues as L-MeAla, D-Leu (×2), D-MeLeu, L-Thr, L-Me-Val, L-Me-Ile, D-Me-*allo*-Ile, D-Val and L-Val. This result left the ambiguity on the localization of the two methylisoleucine residues and of the two enantiomeric valine residues. Since the ^1^H and ^13^C NMR resonances for the amino acids at positions 5–10 and 36–41 of **2** were almost superimposable with the corresponding values reported for theonellapeptolide Id (**1**) [22], we assumed that the two peptides share the configurations at those positions, thus inferring the configurations of the two isoleucine residues. Similarly, it appeared reasonable to assume that, as in the co-occurring theonellapeptolide Id (and in all the theonellapeptolide found to date), the L-Val could be the *N*-terminal amino acid and thus, the D-Val should be the amino acid at positions 28–32. A D-Val residue at these positions has been found previously in the structure of theonellapeptolide Ia [23]. The configuration at the stereogenic sulfur atom of **2** has been left undetermined.

Theonellapeptolide If (**3**, [α]_D_ −26.7, *c* 1.0, MeOH) was isolated as a colorless amorphous solid and its molecular formula was established to be C_68_H_121_N_13_O_16_ by means of high resolution ESIMS. Inspection of ^1^H and ^13^C NMR spectra of **3** (CD_3_OD, Table 2) revealed extensive similarities with parallel data detected for **2**. The most important differences could be recognized in the downfield shift of one methyl singlet (from δ_H_ 2.79, δ_C_ 39.2 in **2** to δ_H_ 3.38, δ_C_ 59.1 in **3**) and of a pair of mutually coupled doublets (from δ_H_ 3.67 and 3.86, δ_C_ 58.9 in **2** to δ_H_ 3.88 and 3.97, δ_C_ 71.8 in **3**). These data were easily rationalized with the replacement of the terminal methylsulfinylacetyl group with a methoxyacetyl group, a typical *N*-terminus acylating unit, already found in the theonellapeptolide family.

**Table 2:** ^1^H (500 MHz) and ^13^C (125 MHz) NMR data of theonellapeptolide If (**3**) in CD_3_OD [24].

AA	Position	δ_H_, m, *J* in Hz	δ_C_	AA	Position	δ_H_, m, *J* in Hz	δ_C_

L-Thr	1	–	169.2	β-Ala	33	–	173.2
	2	4.38, m	57.5		34/34’	2.29, m; 2.59, m	36.5
	3	5.15, m	69.3		35/35’	3.10, m; 4.17, m	36.8
	4	1.13, d, 6.5	17.5	L-Me-Ile	36	–	172.5
D-Me-Val	5	–	171.5		37	3.14, m	71.2
	6	4.95, m	62.2		38	2.48, m	36.5
	7	2.27^a^	26.7		39/39’	1.04, m; 1.92^a^	30.2
	8	0.76, d, 7.2	11.5		40	1.00^a^	13.2
	9	0.99, d, 7.2	23.5		41	0.81, d, 6.4	15.3
	6-NMe	3.17, s	31.3		37-NMe	3.35, s	39.9
				D-Leu	42		176.2
D-Leu	11	–	177.0		43	5.09, m	50.6
	12	5.01, m	48.5		44	1.72^a^; 1.25^a^	41.2
	13/13’	1.65^a^, 1.25^a^	40.3		45	1.78^a^	26.6
	14	1.74^a^	24.9		46	1.03^a^	21.0
	15	0.96^a^	11.1		47	1.04^a^	25.0
	16	0.98 d, 7.3	17.2	β- Ala	48	–	175.0
β-Ala	17	–	174.3		49/49’	2.28, m; 2.36, m	39.1
	18/18’	2.23, m 2.31 m	39.5		50/50’	3.14, m; 3.81, m	37.7
	19/19’	3.15, m 3.85, m	38.2	D-Me-Leu	51	–	175.2
L-Me-Ala	20	–	172.5		52	5.18, m	56.4
	21	5.17^a^	58.5		53/53’	1.41^a^; 2.03 m	39.5
	22	1.45, d, 7.1	16.0		54	1.52^a^	27.1
	21-NMe	2.77 s	30.5		55	0.83, d, 6.7	22.1
L-Me-Val	23	–	173.0		56	0.95^a^	24.3
	24	5.02^a^	59.4		52-NMe	3.30 s	33.0
	25	2.40, m	30.2	L-Val	57	–	176.0
	26	0.86^a^	20.6		58	4.98, m	54.6
	27	0.92, d, 6.1	20.8		59	2.08, m	31.6
	24-NMe	3.31 s	31.9		60	0.98^a^	20.4
D-Val	28	–	173.2		61	1.01^a^	19.2
	29	4.76, m	68.9	MeO-Ac	62		169.2
	30	2.68, m	27.6		63/63’	3.88, d, 11.2;	71.8
	31	0.86^a^	18.1			3.97, d, 11.2	
	32	1.17, d, 6.5	23.3		63-OMe	3.38	59.1

^a^Overlapped with other signals.

Careful inspection of COSY and TOCSY spectra revealed also that, in agreement with the molecular formula, one isoleucine residue of **2** is replaced by a valine residue in **3**. A subsequent combined analysis of homonuclear and heteronuclear 2D NMR spectra disclosed the presence of the following residues: three β-Ala, three Leu (one *N*-methylated), one *N*-methyl-Ile, four Val (two *N*-methylated), one Thr, and one *N*-methylAla. As detailed for **2**, the amino acid sequence of **3** was deduced by careful analysis of HMBC correlations and supported by the MS/MS spectrum of the methanolysis product mixture (see Supporting Information File 1). Thus, theonellapeptolide If (**3**) has been identified as a new member of the theonellapeptolide family differing from theonellapeptolide Id (**1**) by the replacement of two isoleucine residues (positions 5–10 and 28–32) with valine residues. Complete acid hydrolysis of **3** and Marfey’s analysis [21] on the hydrolysate mixture provided the configuration of the chiral amino acid residues as L-MeAla, D-Leu (×2), D-MeLeu, L-Thr, L-Me-Ile, D-Me-Val, D-Val, L-Me-Val and L-Val. With these data in hands, it was not possible to unambiguously define the exact localization of the valine residues. However, if we assume that the L-Val and L-Me-Val residues are localized at the same positions as in theonellapeptolide Id, then the positions of their enantiomeric counterparts are consequently deduced. In summary, compound **3** is a valine-rich theonellapeptolide, being the first member of this class to possess four valine residues.

Theonellapeptolides are structurally similar to the widely used immunosuppressant drug cyclosporin A, sharing at least three important features: i) the exclusive presence of hydrophobic aliphatic amino acids; ii) the abundance of *N*-methylated residues and iii) the presence of a macrocycle made up by more than ten amino acids. Only a few theonellapeptolides have been tested for immunosuppressive activity and they revealed a moderate potency [25]. In general, the potential bioactivity of this class of unique cyclopeptides has not been investigated in great detail, and an inhibition of the development of fertilized eggs of sea urchin [26], an activity on the transport of Na^+^ and K^+^ ions [23], and a moderate cytotoxicity [23] have been reported.

**Figure 4:**
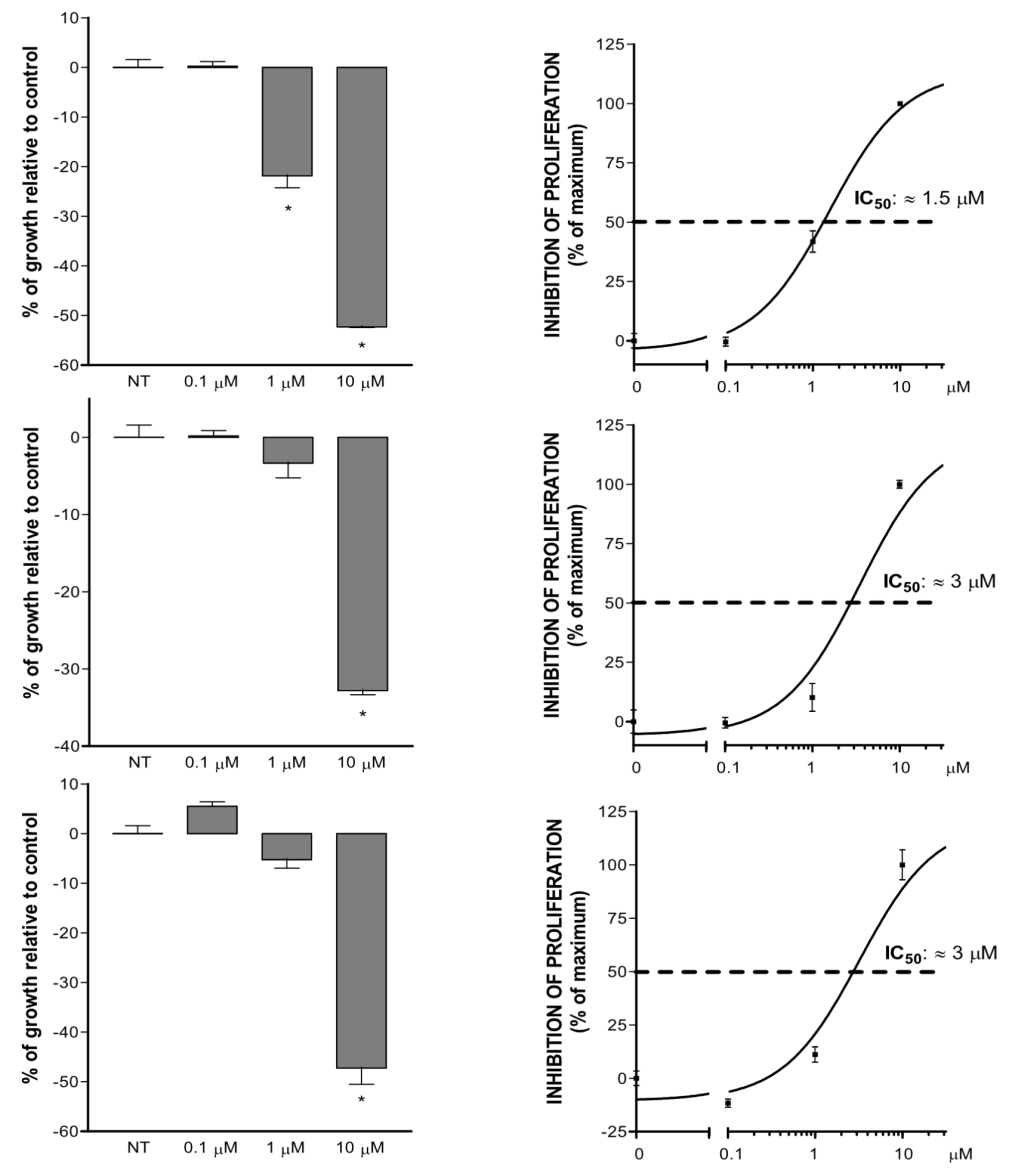
Antiproliferative activity of theonellapeptolides **1**–**3** on hepatic carcinoma cell line. The MTT assay was performed on HepG2 cells treated with increasing doses for 48 hours. Left panel: Proliferation rate expressed as Δ% of absorbance compared to untreated cells. The values are expressed as the mean ± standard error. Right panel: Computation of IC_50_ values. From top to bottom: Theonellapeptolide Id, sulfinyltheonellapeptolide and theonellapeptolide If (* p < 0.05 compared to untreated cells; *n* = 4).

The three theonellapeptolides isolated during the present investigation (**1**–**3**) have been evaluated for their antiproliferative activity against HepG2 cells, a hepatic carcinoma cell line. As reported in Figure 4, all tested compounds showed antiproliferative activity at low micromolar doses, with a similar pattern of potency. At the dose 10 μM, the proliferation rate of HepG2 cells was significantly reduced by theonallapeptolide Id (**1**), sulfinyltheonellapeptolide (**2**) and theonellapeptolide If (**3**) to about 50%, 30% and 50%, respectively (Figure 4, panels A, B and C; * p<0.05 compared to untreated cells; *n* = 4). Only theonellapeptolide Id was able to significantly reduce the proliferation at 1 μM. At this dose, the proliferation reduction was equal to 20% (Figure 4, panel A; * p < 0.05 compared to untreated cells; *n* = 4). Computation of 50% inhibitory drug concentration (IC_50_) revealed that compounds **1**, **2** and **3** were characterized by very similar IC_50_ values of about 1.5 μM for theonellapeptolide Id and 3 μM for sulfinyltheonellapeptolide and theonellapeptolide If.

## Conclusion

In conclusion, the chemical analysis of the organic extract of an Indonesian *Theonella swinhoei* allowed the isolation of two new members of the theonellapeptolide family, namely sulfinyltheonellapeptolide, characterized by a methylsulfinylacetyl group at the *N*-terminus, and theonellapeptolide If, the first member of this class to show four valine residues. The structures of the compounds have been determined by extensive 2D NMR and MS/MS analyses followed by the application of Marfey’s method. The position of D- and L-residues was also assigned on the basis of a comparison with known compounds.

A moderate antiproliferative activity on a hepatic carcinoma cell line has been disclosed for the isolated compounds. A more detailed evaluation of the pharmacological profile of this class of marine peptides is underway in our groups.

## Experimental

**General experimental procedures**: Optical rotations were measured on a Perkin–Elmer 243 B polarimeter. Low and high-resolution ESIMS and MS/MS experiments were carried out on a LTQ OrbitrapXL (Thermo Scientific) mass spectrometer. NMR spectra were obtained on Varian Inova 500 MHz spectrometer (^1^H at 500 MHz, ^13^C at 125 MHz); δ (ppm), *J* in Hz, spectra referred to CD_3_OD (δ_H_ 3.32, δ_C_ 55.0) as an internal standard. Homonuclear ^1^H connectivities were determined by COSY experiments. Single-bond heteronuclear ^1^H/^13^C connectivities were determined with the HSQC experiment. Two and three bond ^1^H/^13^C connectivities were determined by gradient 2D HMBC experiments optimized for a ^2,3^*J* = 9 Hz. Medium-pressure liquid chromatography was performed on a Büchi apparatus by using a silica gel (230–400 mesh) column. HPLC was achieved on a Knauer apparatus equipped with a refractive index detector and analytical LUNA (Phenomenex) columns. The purities of compounds were determined to be greater than 95% by HPLC.

**Animal material, extraction and isolation:*** Theonella swinhoei* (order Lithistida, family Theonellidae) was collected in the Bunaken Marine Park of Manado (North Sualwesi, Indonesia) in January 2010 and frozen immediately after collection. A reference sample of the sponge has been deposited at the Department of Pharmacy, University of Naples Federico II, with the code Man-10-06. The frozen material (16.5 g) was extracted with methanol (3 × 1.5 L) at room temperature, and the crude methanolic extract was subjected to a modified Kupchan’s partitioning procedure as described in [26]. The CHCl_3_ extract (4.76 g) was chromatographed with a silica gel MPLC by using a solvent gradient system from CH_2_Cl_2_ to CH_2_Cl_2_/MeOH 8:2. The fractions eluted with CH_2_Cl_2_/MeOH 97:3 (853.3 mg) were further purified by silica gel column chromatography followed by HPLC on a Nucleodur 100-5 C_18_ (Phenomenex) (5 μm; 7.8 mm i.d. × 250 mm) with MeOH/H_2_O (9:1) as an eluent (flow rate 0.9 mL/min) to give theonellapeptolide Id (**1**) (68.5 mg), sulfinyltheonellapeptolide (**2**, 4.6 mg) and theonellapeptolide If (**3**, 2.1 mg).

**Sulfinyltheonellapeptolide (2):** Colorless amorphous solid; [α]_D_^25^ −38.0 (*c* 0.1, CH_3_OH); ^1^H and ^13^C NMR data in CD_3_OD are given in Table 1; ESIMS: *m*/*z* 1423 [M + H]^+^ and 1445 [M + Na]^+^. HRMS–ESI (*m*/*z*): [M + Na]^+^ calcd for C_69_H_123_N_13_NaO_16_S, 1444.8829; found, 1444.8834.

**Theonellapeptolide If (3):** Colorless amorphous solid; [α]_D_^25^ −26.7 (*c* 0.1, CH_3_OH); ^1^H and ^13^C NMR data in CD_3_OD are given in Table 2; ESIMS (*m*/*z*): 1377 [M + H]^+^ and 1399 [M + Na]^+^. HRMS–ESI (*m*/*z*): [M + Na]^+^ calcd for C_69_H_121_N_13_NaO_16_, 1398.8952; found, 1398.8949.

**Acid hydrolysis and Marfey’s analysis**: Peptide samples (600 μg) were subjected to acid hydrolysis and subsequent Marfey’s analysis in a similar manner as described in [27]. LC–MS analysis (ESIMS, positive ion mode): individual FDAA-amino acid peak was identified by co-injection with standard amino acid derivatives. The retention times (min) of the FDAA standards were 18.15 for L-Thr, 21.50 for L-MeAla, 29.91 for l-Val, 34.04 for L-MeVal, 34.23 for D-Val, 38.25 for L-MeIle, 34.42 for D-MeVal, 41.35 for D-*allo*-MeIle, 43.20 for D-Leu, 45.08 for D-MeLeu. Observed amino acids and their retention times (min) for FDAA derivatives of acid hydrolysate of **2** and **3**: **2** 18.12 for L-Thr, 21.39 for L-MeAla, 29.98 for l-Val, 34.01 for L-MeVal, 34.31 for D-Val, 38.18 for L-MeIle, 41.32 for D-*allo*-MeIle, 43.19 for D-Leu, 45.01 for D-MeLeu. **3** 18.12 for L-Thr, 21.36 for L-MeAla, 29.99 for l-Val, 34.02 for L-MeVal, 34.29 for D-Val, 38.19 for L-MeIle, 34.40 for D-MeVal, 43.17 for D-Leu, 45.02 for D-MeLeu.

**Evaluation of antiproliferative activity:** HepG2 cells were plated in a 24-wells plate at 3 × 10^4^ cells/well, in Minimum Essential Medium with Earl's salts containing 10% fetal bovine serum (FBS), 1% L-glutamine and 1% penicillin/streptomycin. On day 2, cells were treated with increasing doses of theonellapeptolide Id (0.1, 1 and 10 μM), sulfinyltheonellapeptolide (0.1, 1 and 10 μM) and theonellapeptolide If (0.1, 1 and 10 μM) for 48 hours. On day 4, a MTT assay was assessed: 100 μL of MTT solution (5mg/mL) were added to each well and the cells were incubated at 37 °C for 4 hours. After the incubation, the culture medium was removed and 1 mL of DMSO was added to each well. The absorbance was read by using a spectrophotometer at 590 nM. The proliferation rate was reported as the delta of absorbance compared to untreated cells, and values are expressed as mean ± standard error. Experiments were performed in quadruplicate. For each compound the IC_50_ value was evaluated as follows: The inhibition of proliferation was expressed as percentage compared to the inhibition of proliferation observed at the highest dose of any experimental setting (which was arbitrarily assigned a value of 100%).

## Supporting Information

File 1 COSY, key HMBC correlations and MS/MS fragmentations of **3**, and ^1^H and COSY spectra for sulfinyltheonellapeptolide (**2**) and theonellapeptolide If (**3**).
