# Supplementary material for: New tridecapeptides of the theonellapeptolide family from the Indonesian sponge Theonella swinhoei
Source: Beilstein J Org Chem. 2013 Aug 13;9:1643–51. doi: 10.3762/bjoc.9.188 (PMC3778368; doi:10.3762/bjoc.9.188)
Supplement: File 1 — COSY, key HMBC correlations and MS/MS fragmentations of 3, and 1H and COSY spectra for sulfinyltheonellapeptolide (2) and theonellapeptolide If (3). [file Beilstein_J_Org_Chem-09-1643-s001.pdf]

Supporting Information

for

New tridecapeptides of the theonellapeptolide family from the Indonesian sponge *Theonella swinhoei*

Annamaria Sinisi[1], Barbara Calcinai[2], Carlo Cerrano[2], Henny A. Dien[3], Angela Zampella[1], Claudio D'Amore[4], Barbara Renga[4], Stefano Fiorucci[4] and Orazio Taglialatela-Scafati[1,*,§]

Address: [1]Dipartimento di Farmacia, Università di Napoli "Federico II", via D. Montesano 49, 80131 Napoli, Italy, [2]Dipartimento di Scienze della Vita e dell'Ambiente, Università Politecnica delle Marche, Via Brecce Bianche, 60131 Ancona, Italy, [3]Faculty of Fishery and Marine Science, Sam Ratulangi University, Manado, Indonesia and [4]Dipartimento di Medicina Clinica e Sperimentale, Università di Perugia, Via Gambuli 1, 06132 Perugia, Italy

Email: Orazio Taglialatela-Scafati - scatagli@unina.it

*Corresponding author

[§]Tel.: (0039) 081-678509, Fax (0039) 081-678552

COSY, key HMBC correlations and MS/MS fragmentations of 3, and ^{1}H and COSY spectra for sulfinyltheonellapeptolide (2) and theonellapeptolide If (3)

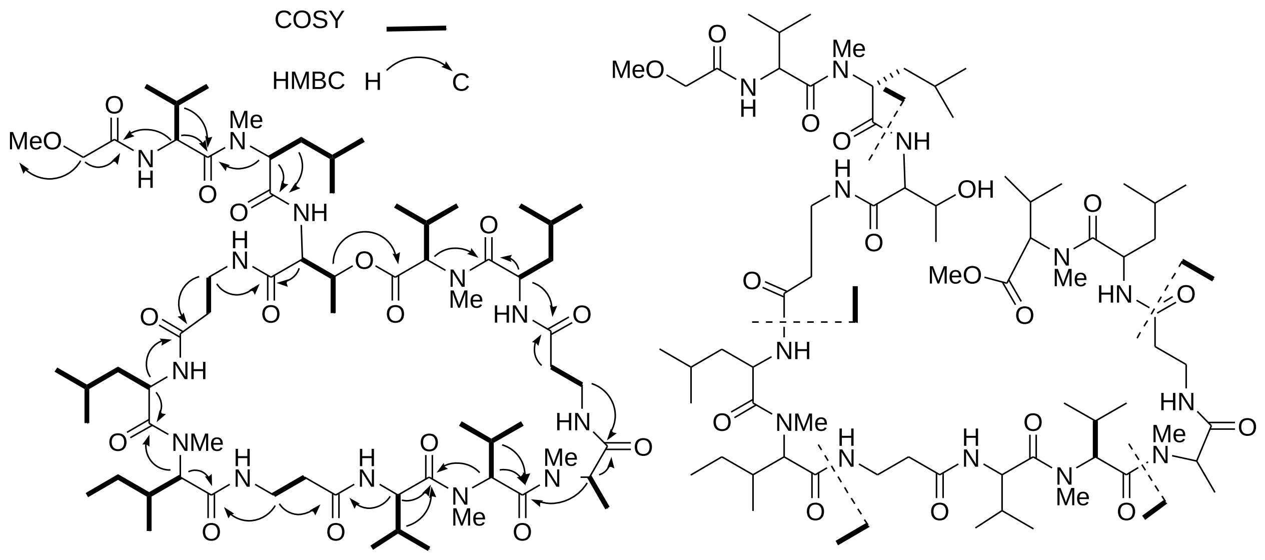

Figure S1: COSY and key KMBC correlations (left) and MS/MS fragmentations of **3**.

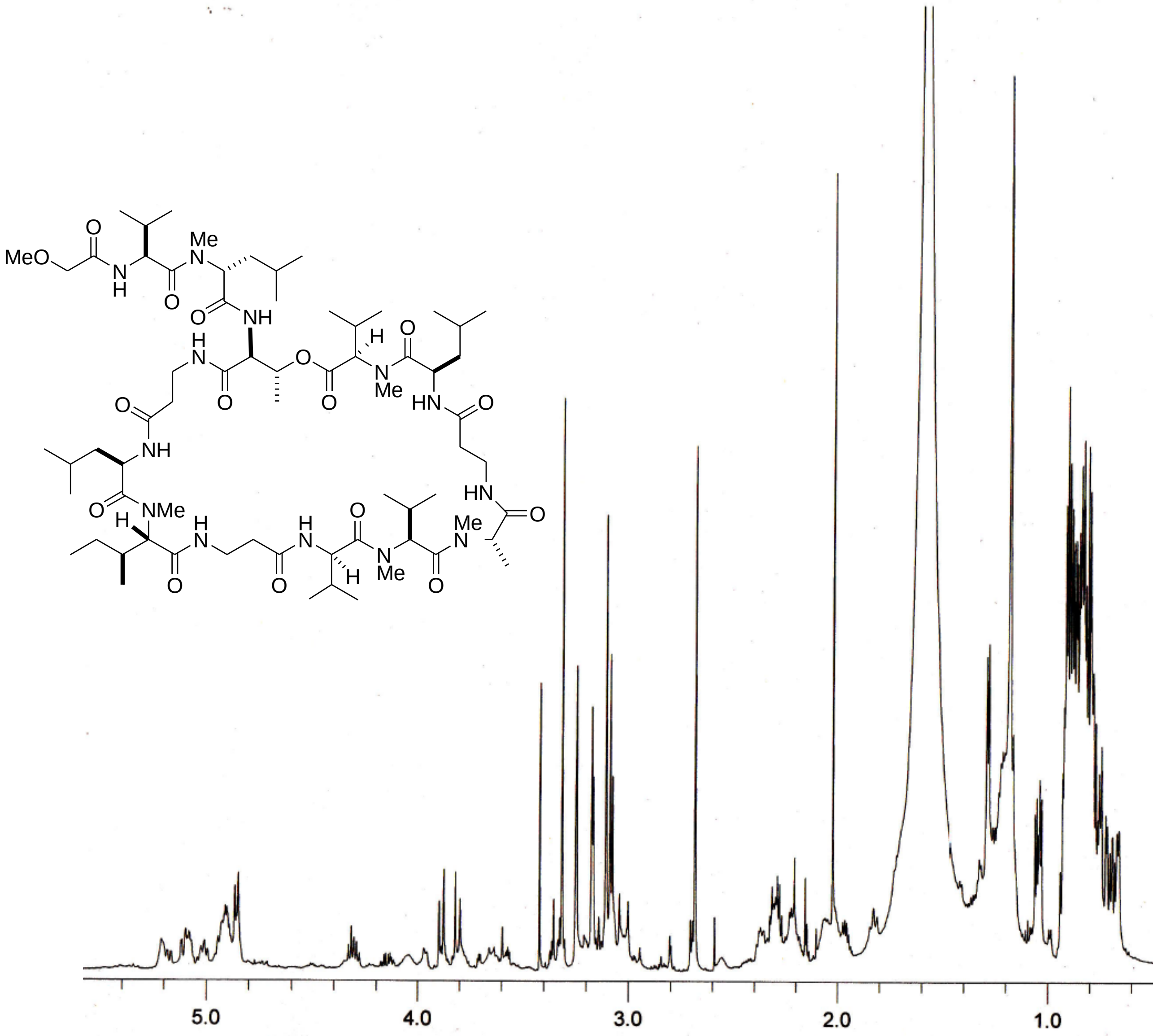

Figure S2: ^{1}H NMR spectrum (CD_3OD) of theonellapeptolide If.

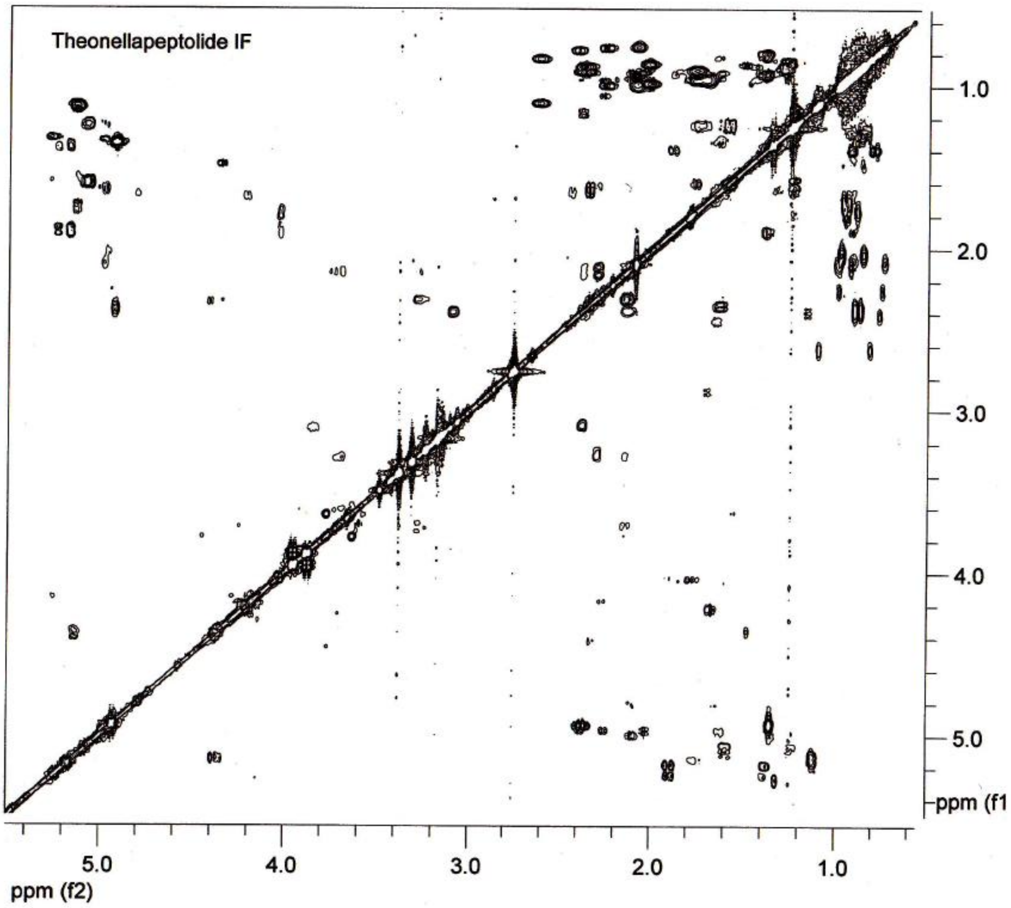

Figure S3: COSY NMR spectrum (CD_3OD) of theonellapeptolide If.

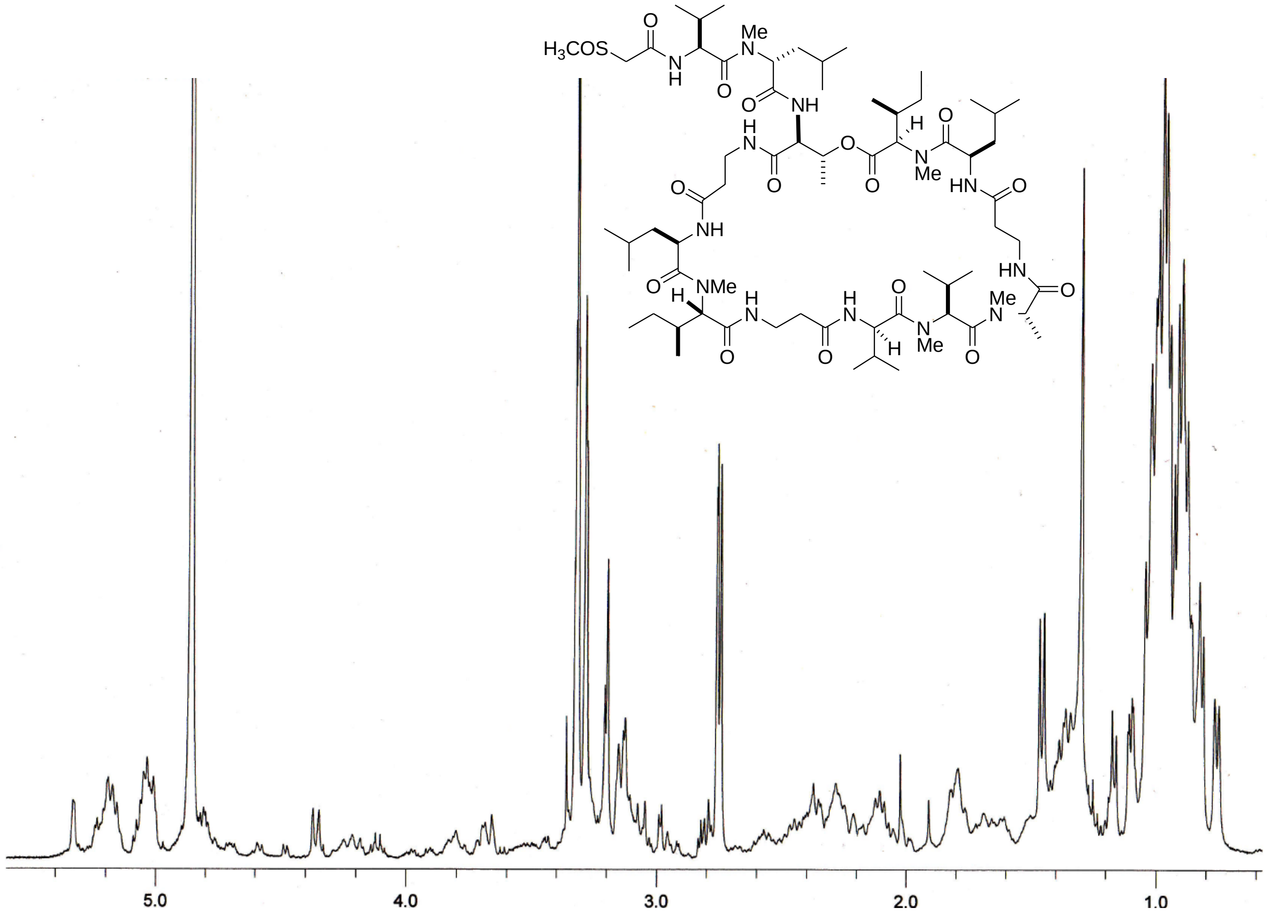

Figure S4: ^{1}H NMR spectrum (CD_3OD) of sulfinyltheonellapeptolide.

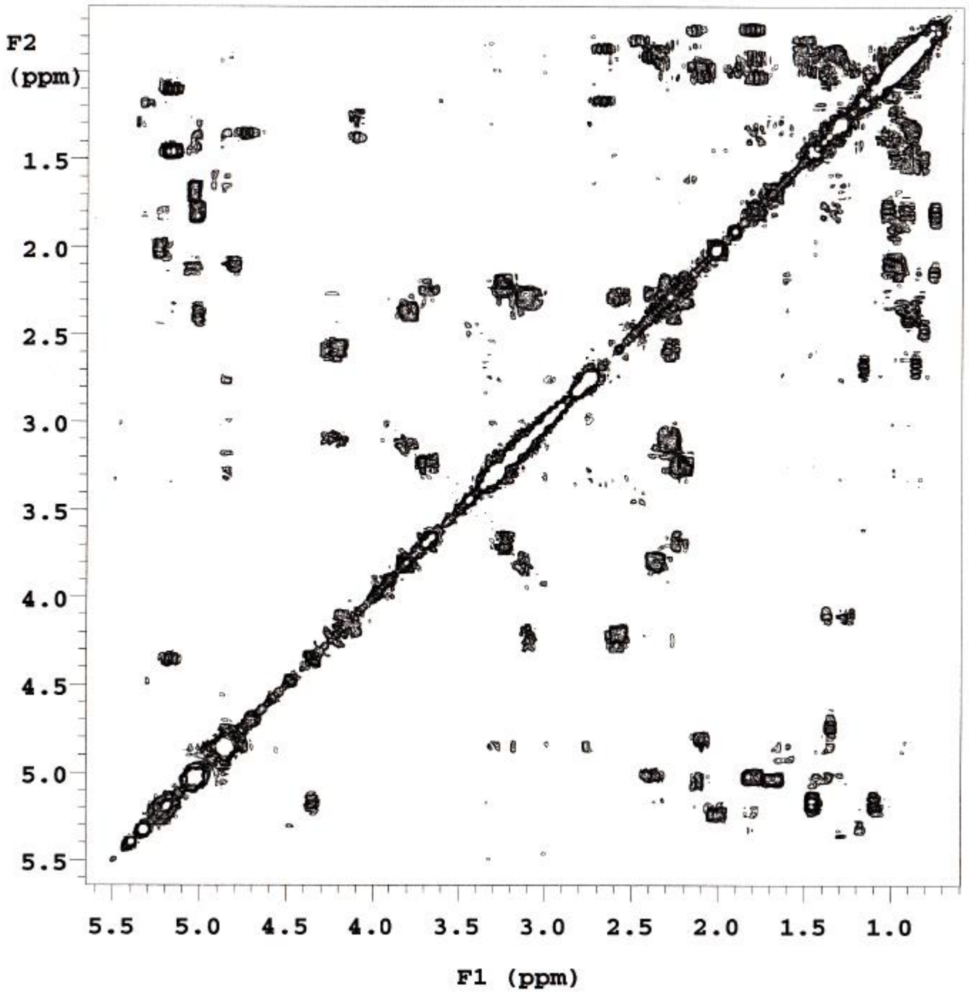

Figure S5: COSY NMR spectrum (CD_3OD) of sulfinyltheonellapeptolide.
